# Performance of comprehensive cardiac magnetic resonance imaging including T1 and T2 mapping on 1.5 vs. 3.0 Tesla as compared to biventricular endomyocardial biopsy in patients with suspected myocarditis - the MyoRacer trial

**DOI:** 10.1186/1532-429X-18-S1-O98

**Published:** 2016-01-27

**Authors:** Philipp Lurz, Christian F Luecke, Marcus Sandri, Enno Boudriot, Ingo Eitel, Suzanne de Waha, Karin Klingel, Reinhard Kandolf, Matthias Grothoff, Gerhard Schuler, Holger Thiele, Matthias Gutberlet

**Affiliations:** 1 Cardiology, Heart Center of the University Luebeck, Leipzig, Germany; 2 Department of Radiology, Heart Center Leipzig, Leipzig, Germany; 3 Internal Medicine/Cardiology, Heart Center of the University Leipzig, Leipzig, Germany; 4 Department of Molecular Pathology, University Hospital Tuebingen, Tuebingen, Germany

## Background

The diagnostic performance of cardiac magnetic resonance (CMR) imaging in suspected myocarditis is still limited. Recently, CMR T1 and T2 mapping has been suggested to yield excellent diagnostic accuracies in patients with suspected myocarditis as compared to healthy controls. However, the true diagnostic performance of CMR mapping when compared to endomyocardial biopsy (EMB) and the impact of CMR field strength in patients with a variety of pathologies is still unknown and was therefore assessed in this study.

## Methods

Within the final analyses, 129 consecutive patients with suspected acute or chronic myocarditis were included. Patients had to fulfill indications for CMR imaging according to the JACC White consensus paper.

All patients underwent biventricular EMB, cardiac catheterization for exclusion of coronary artery disease, and CMR on a 1.5 (Intera, CV, Philips Medical Systems, Best, The Netherlands) and 3.0 (Magnetom Verio, Siemens Healthcare, Erlangen, Germany) Tesla tomography.

The CMR protocol included standard Lake-Louise (LL) parameters as well as native and post contrast T1 and T2 mapping. Relative Enhancement was assessed with a T1 weighted TSE sequence, edema ratio with a T2 weighted STIR sequence. T1 Mapping was measured with a MOLLI-Sequence pre and post contast (0.015 mmol Gadobutorol per kg body weight (Gadovist, Bayer Healthcare, Leverkusen, Germany) and T2 Mapping with a Multi Echo Spin Echo sequence on the 1.5T scanner and a T2 prepared SSFP sequence on the 3T scanner. Patients were divided into 2 groups according to duration of symptoms: patients with acute symptoms (≤14 days) and those with chronic symptoms (>14 days).

## Results

The diagnostic performance of LL criteria, native T1 and extracellular volume fraction (ECV) as well as T2 mapping is summarized in the table. On 1.5 Tesla, in patients with acute symptoms, native T1 yielded best diagnostic performance (81%) followed by ECV (75%) and T2 (72%). In chronic patients, T2 was the only technique achieving an acceptable accuracy (72%). Accuracies of imaging techniques on 3.0 Tesla CMR were slightly lower as compared to 1.5 Tesla. However, T2 mapping on 3.0 Tesla appeared to be unsatisfactory for the diagnosis of myocarditis.

## Conclusions

In patients with acute symptoms and suspected myocarditis, mapping techniques provides a useful tool for the confirming or rejecting the diagnosis of myocarditis and are superior to LL criteria. In contrast, only T2 mapping results in an acceptable diagnostic accuracy in patients with chronic symptoms. The impact of field strength on diagnostic accuracies requires further exploration.

**Figure 1:**
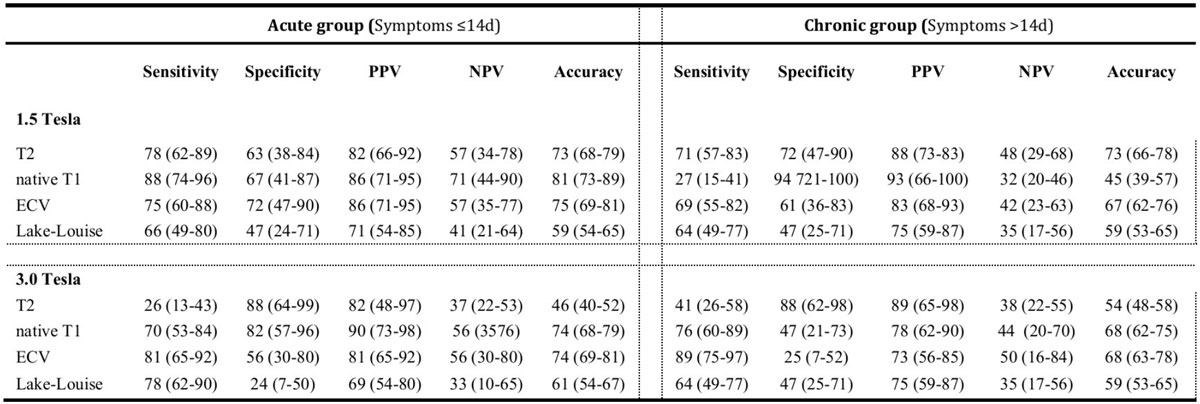
Figure 1

